# Vitamin D_3_ supplementation of a high fat high sugar diet ameliorates prediabetic phenotype in female LDLR^−/−^ and LDLR^+/+^ mice

**DOI:** 10.1002/iid3.154

**Published:** 2017-03-13

**Authors:** Ramiar Kheder, James Hobkirk, Zeayd Saeed, Justyna Janus, Sean Carroll, Michael J. Browning, Cordula Stover

**Affiliations:** ^1^ Department of InfectionImmunity and InflammationUniversity of LeicesterLeicesterUK; ^2^ College of NursingUniversity of RaparinKurdistan RegionIraq; ^3^ Department of SportHealth and Exercise ScienceUniversity of HullHullUK; ^4^ Department of NursingTechnical institute of SamawaIraq; ^5^ Preclinical Imaging FacilityCore Biotechnology ServicesUniversity of LeicesterLeicesterUK; ^6^ Department of ImmunologyLeicester Royal InfirmaryLeicesterUK

**Keywords:** Diet, metabolic syndrome, mouse study, Vitamin D

## Abstract

**Introduction:**

Fatty liver disease is prevalent in populations with high caloric intake. Nutritherapeutic approaches are being considered, such as supplementary Vitamin D_3_, to improve aspects of metabolic syndrome, namely fatty liver disease, hyperlipidemia, and insulin resistance associated with obesity.

**Methods:**

We analyzed female LDLR^−/−^ and LDLR^+/+^ mice on a 10‐week diabetogenic diet for markers of fatty liver disease, metabolic strain, and inflammation.

**Results:**

The groups on a high fat high sugar diet with supplementary Vitamin D_3_, in comparison with the groups on a high fat high sugar diet alone, showed improved transaminase levels, significantly less hypertriglyceridemia and hyperinsulinemia, and histologically, there was less pericentral hepatic steatosis. Levels of non‐esterified fatty acids and lipid peroxidation products were significantly lower in the group supplemented with additional Vitamin D_3_, as were systemic markers of inflammation (serum endotoxin and IL‐6). M2 macrophage phenotype predominated in the group supplemented with additional Vitamin D_3_. Beneficial changes were observed as early as five weeks’ supplementation with Vitamin D_3_ and extended to restoration of high fat high sugar diet induced decrease of bone mineral density.

**Conclusion:**

In summary, Vitamin D_3_ was a significantly beneficial dietary additive to blunt a prediabetic phenotype in diet‐induced obesity of female LDLR^−/−^ and LDLR^+/+^ mice.

## Introduction

Obesity is a serious health problem in industrialized countries. It associates with chronic diseases and cancer 1. Selective consumption of so‐called “energy dense foods,” those rich in fats and sugars, is responsible for a significant increase in body weight in the population 2. In man, “metabolic syndrome” describes a complex manifestation of obesity, encompassing hypertension, dyslipidaemia, “non‐alcoholic” fatty liver disease (NAFLD), and development of diabetes. NAFLD is strongly associated with obesity and its metabolic complications and comprises a disease spectrum ranging from simple steatosis (fatty liver), through non‐alcoholic steatohepatitis (NASH), characterized by excessive liver inflammation, to fibrosis. Diabetes develops from insulin resistance, a relative insufficiency of insulin‐mediated decrease of blood glucose levels.

Mouse models of dietary‐induced obesity, including NAFLD development, appear to adequately model several of the metabolic abnormalities and heterogeneity evident in the human metabolic syndrome 3. Notably, C57BL/6J mice given a high fat, high carbohydrate diet for four months exhibited severe pan‐lobular steatosis (in the absence of an inflammatory component), a marked increase in hepatic triglyceride levels, and profound peripheral insulin resistance 4.

The first mouse genetic model of familial hyperlipidemia, the Low‐density lipoprotein (LDL) receptor knockout mouse (LDLR^−/−^), has not been widely considered for characterisation of the metabolic syndrome and complications. However, the LDLR^−/−^ mouse model has been viewed as useful when studying diet‐induced obesity and insulin resistance in the presence of hyperlipidemia 5. Early studies 6 showed LDLR^−/−^ mice fed a Western‐type diet (high fat and/or high sugar) developed fatty liver and characteristics of non‐alcoholic steatohepatitis and these findings have since been replicated. Longer term Western‐style diet feeding, especially in the presence of oxidative stressors such as dietary cholesterol, induces a severe NASH phenotype in LDLR^*−/−*^ mice with histological and biochemical evidence of steatosis, inflammation (with endotoxaemia), and hepatic fibrosis 7, 8. Based on the expression of these features of metabolic syndrome, the mouse model appears representative of the phenotype of NASH in obese humans with metabolic syndrome.

Supplementation of a high fat‐olive oil–containing diet with fish oil was shown to have an antiinflammatory effect in LDLR^*−/−*^ mice, as evidenced by reduced macrophage infiltration and inflammatory gene expression in white adipose tissue 9. This was associated with reduced liver and plasma lipids. However, other specific dietary interventions (low fat, low cholesterol) were insufficient to fully reverse NASH phenotype expression in male LDLR^*−/−*^ mice after eight months’ Western Diet 10.

A large prevention study revealed a reduced risk of developing metabolic syndrome with an increase in Vitamin D levels 11. Vitamin D_3_ exerts an anti‐inflammatory effect 12. The potential of Vitamin D_3_ supplements to ameliorate aspects of disease has been studied in clinical trials: However, a conclusive, beneficial treatment effect of Vitamin D_3_ supplementation was neither found in NAFLD 13, nor in diabetes 14, nor in lipid profile 15, but doses and duration of Vitamin D_3_ supplementation varied widely.

Kitson and Roberts 16 have indicated that Vitamin D deficiency also closely relates to the severity of NAFLD and is implicated in the pathogenesis of insulin resistance, a key factor in the development of NAFLD. Inflammation has been implicated as a contributing factor to dysregulated hepatic insulin signaling 17 and steatosis. The anti‐inflammatory and immune‐modulatory properties of Vitamin D are putative mechanisms of its effect on liver disease progression.

Our previous in vitro study demonstrated a significant effect of Vitamin D_3_ in decreasing lipopolysaccharide (LPS)‐induced production of TNFα and TGFβ from macrophages and hepatocytes 18, and justified analyzing the efficacy of dietary supplementation with Vitamin D_3_ to improve biochemical and inflammatory features of metabolic disease in vivo. So far, only few in vivo studies using mice have evaluated the effects of dietary Vitamin D_3_ on measures of inflammation and metabolism: in male C57Bl/6J mice given a high fat diet (HFD) (60% energy as fat) on its own or with tenfold the recommended dose of Vitamin D_3_ (1000 IU/kg) and an additional (small) oral dose of Vitamin D_3_ in soybean oil (25 IU/mouse/day) for 10 weeks, hyperinsulinemia in the HFD group was normalized in the group receiving Vitamin D_3_, while adiponectin was significantly elevated in the Vitamin D_3_ group compared to the HFD group 19. The rise in adiponectin is consistent with an increase in insulin sensitivity 20. Similarly, in male Swiss mice fed a HFD (20% of fat from soy beans) for four weeks, then split into two groups of which one was given Vitamin D_3_ supplement (0.05 mg/kg diet) for an additional four weeks, the Vitamin D_3_ supplemented group showed a decrease in IL‐6 protein and increase of IL‐10 protein in epididymal adipose tissue, an inflammatory signature of an increase in insulin sensitivity 21. When male LDLR^−/−^ mice were given a diabetogenic diet, consisting of 35.5% sugar and 36.6% fat for 24 weeks, they developed hepatomegaly, expressed more TNFα, IL‐6 and MCP‐1 mRNA in their livers, had elevated levels of insulin, transaminases, and hepatic triglycerides when compared to their controls 22.

The aim of this study was to determine the effect of one dose of dietary Vitamin D_3_ supplementation on liver transaminase levels, endotoxin levels, as well as inflammatory mediators and metabolic parameters for the duration of ten weeks and to probe for an effect as early as five weeks in female C57Bl/6 mice and LDLR^−/−^ mice.

## Materials and Methods

### Experimental design

Animal experimentation was performed in accordance with UK Home Office regulations and institutional guidelines. The study was periodically reviewed by the ethical review body of the institution. Mice were housed in a specific pathogen free barrier facility in groups in ventilated cages at 21°C, 50% humidity, with 12/12 h light/dark cycle, and had ad libitum access to food and water. Mice were maintained on 5LF2 (14% protein, 6% fat, 65% carbohydrate), so‐called maintenance diet, containing 1 IU/g Vitamin D_3_. At three months’ of age, female LDLR^−/−^ and LDLR^+/+^ were randomized to two groups fed ad libitum for five or ten weeks the formulated (cholesterol free) diet 58R3 (20% protein, 36% fat, 35% carbohydrate, rich in sucrose), termed DD (diabetogenic diet) differing in the content of admixed Vitamin D_3_ (1 vs. 11 IU/g diet) (TestDiet, International Product Supplies, London, UK). Diets were color coded and food was replaced weekly. Sixty mice were used in total and analysed in littermate groups. Gnawing blocks were added to the cage floor covered with corn cob as bedding material; nesting material (sizzle pet) was made from recycled paper. Because male mice behaved aggressively, leading to diet unrelated variation in energy expenditure, female mice were taken for a more homogenous analysis of a response to the diet. There was equal environmental enhancement for all. Mice were weighed weekly and handled by the same person. At the end of study, mice were bled under terminal anesthesia, serum prepared, and organs saved for further measurements. Analyses were conducted blinded to the genotypes and treatment. Liver weights were recorded and expressed as % of body weight. LDLR^−/−^ and the equally in house bred background strain, C57Bl/6, verified to be LDLR^+/+^ (by genotyping), was used in comparison.

### Measurement of metabolic parameters

Activities of liver transaminases AST (aspartate aminotransferase) and ALT (alanine aminotransferase) were determined in serum samples diluted 1:5 following the manufacturer's instructions (Abcam, Cambridge, UK). Non‐esterified fatty acids were measured using NEFA Assay kit from WAKO Chemicals GmbH (Neuss, Germany). Triglyceride colorimetric assay kit was from Cayman Chemical (Michigan, USA). Malonedialdehyde and insulin were measured by immunoassay kit (Abcam and Biorbyt, Cambridge, UK). Adiponectin was measured using ELISA Mouse Adiponectin/Acp30 (R&D systems, Abingdon, UK) using serum in 2000‐fold dilution as suggested by the manufacturer. HbA1c was measured in 1:10 diluted serum samples using mouse glycated Hemoglobin A1c ELISA kit as indicated by the supplier (CUSABIO, Wuhan, China). Mouse 1,25‐dihydroxy‐Vitamin D_3_ ELISA kit was from CUSABIO.

### Measurement of inflammatory component

Endotoxin was measured by LAL method in serum diluted 1:50 (Pierce™ LAL chromogenic endotoxin quantification kit, ThermoScientific). Murine IL‐6 ELISA kit (Peprotech, London, UK) was used according to manufacturer's instructions. Serum dilution was 1:10.

### Histopathological assessment

Four micrometer sections were prepared from paraffin embedded specimens and stained with hematoxylin and eosin, and evaluated following published criteria 23.

### qPCR analysis

RNA was prepared using RNeasy Mini Kit (Qiagen, Manchester UK), genomic DNA was digested and cDNA was synthesized using RevertAid H Minus First Strand cDNA Synthesis Kit (Thermo Fisher Scientific, Loughborough, UK). Primer sequences were: for arginase 5′‐GGGAATCTGCATGGGCAAC‐3′, 5′‐GCAAGCCAATGTACACGATGTC‐3′; iNOS 5′‐GGCAGGCCTGTGAGACCTTTG‐3′, 5′‐GAAGCGTTTCGGGATCTGAA‐3′; Srebp‐1c 5′‐TCTGCCTTGATGAAGTGTGG‐3′, 5′‐AGCAGCCCCTAGAACAAACA‐3′; endogenous reference gene (GAPDH) 5′‐CCTGGAGAAACCTGCCAAGTATG‐3′, 5′‐AGAGTGGGAGTTGCTGTTGAAGTC‐3′. GAPDH was checked for stability of expression across the dietary groups before using as endogenous reference gene. The Livak or 2^−ΔΔCT^ method was used to calculate the normalized expression ratio of target gene and reference gene 24.

### Quantification of bone mineral density

Spines from six 3‐month‐old LDLR^+/+^ mice that were fed for five weeks either the maintenance diet or the diabetogenic diet (with or without Vitamin D_3_ supplementation) were studied. Following terminal anesthesia, the spinal columns were dissected and fixed in 10% formal saline. All bone samples were stored in PBS at −4°C. The specimens were scanned with a high speed in vivo μCT scanner (Quantum FX, PerkinElmer Inc.). All images were acquired blinded to the treatment with the following parameters: 90 kVp voltage, 80 μA current, field of view (FOV) 5 × 5 mm, and with isotropic resolution of 10 μm. The bone mineral density for the lumbar vertebra L3 was estimated using a calcium hydroxyapatite (CaHA) phantom (MicroCT‐HA, Quality Assurance in Radiology and Medicine GmbH), scanned using the same parameters as for the spines. For accurate bone density calibrations, images of the phantom consisting of six different CaHA concentrations were analysed. Linear regression of measured grayscale values versus known mineral density of phantom regions (mg HA/cm^3^) was plotted. The obtained SigmaCT and BetaCT values were then used for conversion of X‐ray values to quantify real cortical and trabecular bone density.

Custom software (Analyze 12.0, AnalyzeDirect) was used for semi‐automatic image processing. Firstly, the Volume Edit tool was used to create a segmentation mask. Determination of the optimal threshold that excludes soft tissue but includes only the bone of interest was kept the same for all samples. The created segmentation mask of the bone was loaded onto the bone mineral analysis add‐on for determination of cortical and trabecular bone by their further thresholding and correction steps. The final image was used as a mask to measure mineral density of cortical and trabecular bone of the lumbar spines.

### Statistical analysis

Gaussian distribution of measurements was assumed. Data were presented as means ± SD and analysed by unpaired *t*‐test using Prism Pad 6. A *P*‐value <0.05 was deemed significant.

## Results

The high fat high sugar diet was palatable for the mice. LDLR^+/+^ and LDLR^−/−^ exceeded the normal weight gain of 10–15% over 10 weeks of maintenance diet by about fourfold when on a high fat high sugar diet. At the end of the 10‐week feeding period, body weights of the groups fed the diabetogenic diet (DD) and the groups fed the DD with additional Vitamin D_3_ supplementation, were not significantly different from one another. Their dietary intakes were comparable and furs appeared equally matted.

The dose of Vitamin D_3_ chosen as additive to the high fat high sugar diet was based on a study of potential toxicity of Vitamin D_3_ in mice 25. At a dose of 0.05 mg/kg body weight/d (equivalent to the additional dose admixed to the formulated diet (10 IU/g diet) based on a daily intake of 5 g), mice did not lose weight, maintained stable calcium levels and generated serum levels of Vitamin D_3_ which were significantly below those associated with hypercalcemia and toxicity. The serum levels of Vitamin D_3_ measured in the experimental mice of our study were even lower than these non‐toxic levels previously reported 25. Proof that supplementation with Vitamin D_3_ yielded elevation in blood levels of Vitamin D_3_ is given in supplementary Table S1. The lower levels of Vitamin D_3_ in serum of LDLR^−/−^ compared to LDLR^+/+^ fed the high fat high sugar diet with Vitamin D_3_ supplementation is likely to be reflective of greater binding of Vitamin D_3_ to lipoproteins which are elevated in LDLR^−/−^ 26, 27. Mice were subsequently analyzed for markers of fatty liver disease, metabolic strain, and inflammation, following the hypothesis of a beneficial effect of Vitamin D on key corollaries of a continued high fat high sugar diet.

**Figure 1:**
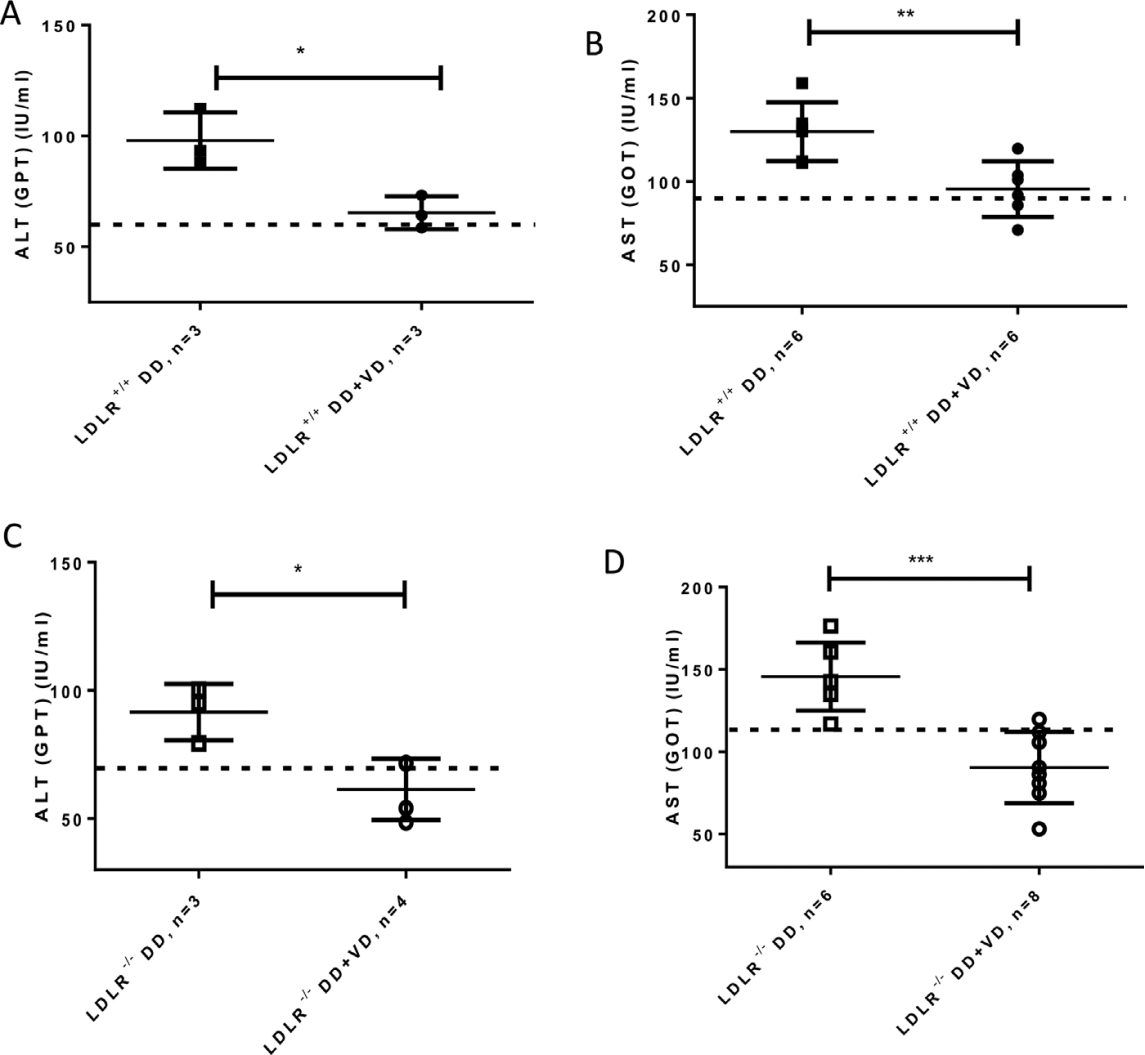
Amelioration of transaminase elevations in LDLR^+/+^ (A and B) and LDLR^−/−^ (C and D) fed a diabetogenic diet with or without admixed Vitamin D_3_ for 10 weeks. Dashed line indicates basal level of activities in LDLR^+/+^ or LDLR^−/−^ fed a normal, maintenance diet. *P 0.05, **P < 0.005, ***P < 0.0005.

At our timepoint of 10 weeks, there was no hepatomegaly, but macroscopic (yellowish discoloration of the liver) and microscopic signs of fatty liver changes. As observed by others, there were stark regional differences in manifestation of fatty liver changes 28. Where these were present, analysis of the hepatic lobular zones revealed that lipid accumulation in hepatocytes occurred near the periportal field in LDLR^+/+^ and importantly, near the central vein in LDLR^−/−^ fed a high fat sugar diet, and was improved in the groups given supplementary Vitamin D_3_ (supplementary Fig. S1). mRNA expression of lipogenic transcription factor Strebp‐1c was significantly decreased in livers from both, LDLR^+/+^ and LDLR^−/−^ fed a high fat sugar diet with supplementary Vitamin D_3_ compared to their controls (supplementary Fig S1), demonstrating transcriptional adaptation to the presence of Vitamin D_3_ in avoidance of developing steatosis. Hepatic transaminases as markers of hepatocellular damage were determined in serum and found to be elevated in mice fed the high fat high sugar diet (compared to mice fed the maintenance diet) but significantly lowered in LDLR^−/−^ and LDLR^+/+^ mice receiving Vitamin D_3_ supplemented high fat high sugar diet (Fig. 1A–D). Importantly, the levels achieved in Vitamin D_3_ supplemented groups were not significantly lower than the levels in mice receiving maintenance diet, meaning that there were no signs of intervention induced hepatotoxicity 29.

**Figure 2:**
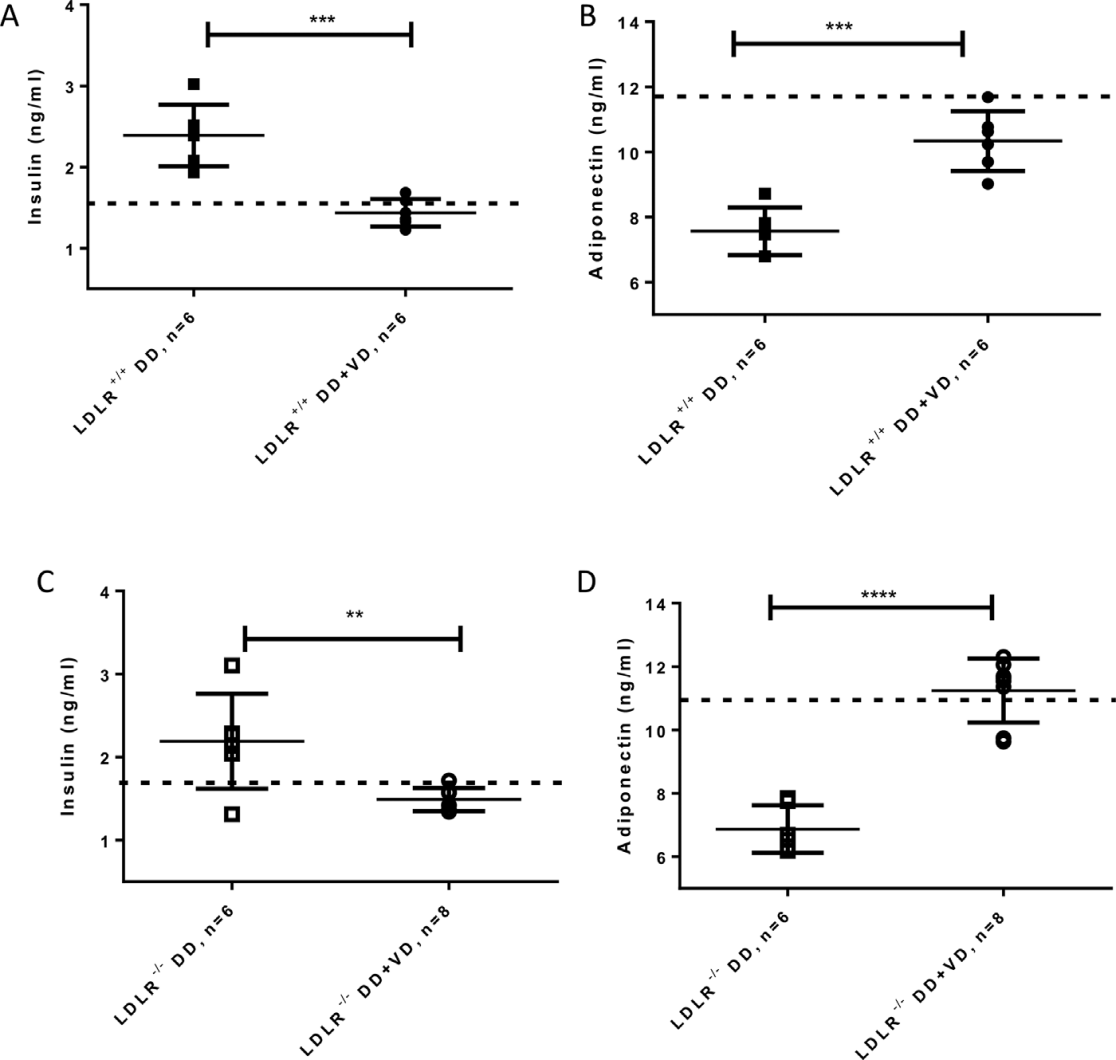
Correction of hyperinsulinemia in the presence of Vitamin D_3_ supplemented to 10 weeks’ diabetogenic diet. Dashed line indicates basal level of hormones in LDLR^+/+^ or LDLR^−/−^ fed a normal, maintenance diet. **P < 0.005, ***P < 0.0005, ****P < 0.0001.

To gauge the extent glucose control was compromised in this model, glucose, insulin, adiponectin, and HbA1c levels were determined. High fat high sugar diet for ten weeks provoked in LDLR^+/+^ and LDLR^−/−^ mice elevated levels of insulin (non fasting blood glucose was not different from normal, data not shown) and inversely, depressed levels of adiponectin, which were both close to normal when additional Vitamin D_3_ was admixed to the diet (Fig. 2A–D). Serum levels of adiponectin in mice on high fat high sugar diet were markedly lower than those in mice on maintenance diet, as expected 30. HbA1c levels allow sensitive appraisal of blood glucose levels over the duration of the 10‐week diet (mouse red blood cells have a life span of 40 days). Vitamin D_3_ supplementation of a high fat high sugar diet reduced aberrant protein glycosylation (HbA1c as surrogate marker) significantly for each genotype (supplementary Table S2).

**Figure 3:**
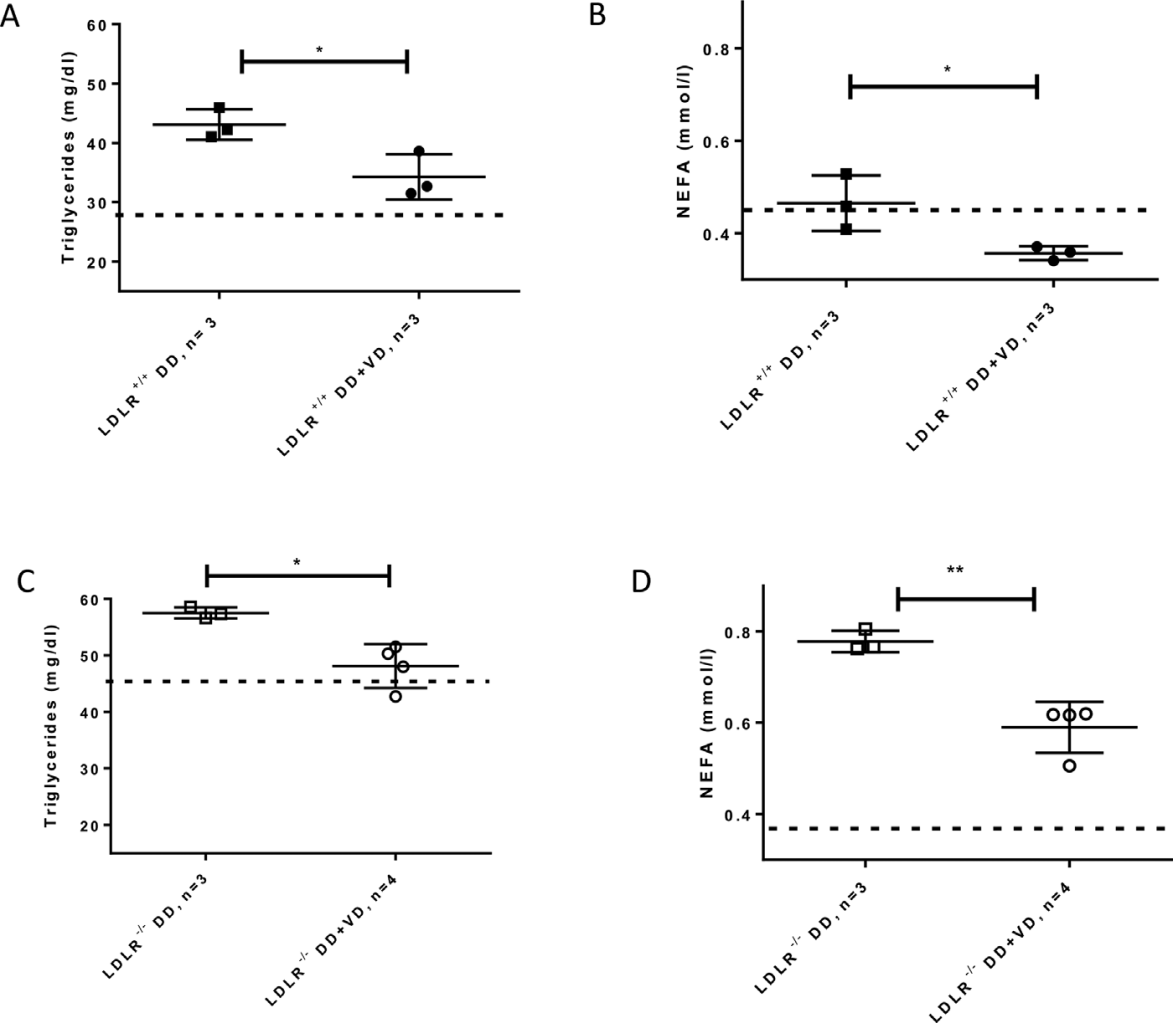
Serum triglycerides and non‐esterified, free fatty acids in LDLR^+/+^ (A and B) and LDLR^−/−^ (C and D) fed a diabetogenic diet without or with additional Vitamin D_3_ for 10 weeks. Dashed lines in graphs represent levels measured in mice fed the maintenance diet, respectively. *P < 0.05, **P < 0.005.

LDLR^−/−^ mice had a higher basal triglyceride level than LDLR^+/+^ mice as expected from their deficiency of LDL receptor and associated defect in lipoprotein clearance (Fig. 3A and C). LDLR^+/+^ and LDLR^−/−^ mice developed significant hypertriglyceridemia at the 10 week‐endpoint of the HFD study. Vitamin D_3_ supplementation of high fat high sugar diet led to normalization of triglyceride levels in both genotypes. Similarly, NEFA levels were higher in diet‐stimulated LDLR^−/−^ compared to LDLR^+/+^ as expected. Vitamin D_3_ supplementation led to significantly reduced levels of NEFA in both genotypes after 10 weeks (Fig. 3B and D).

**Figure 4:**
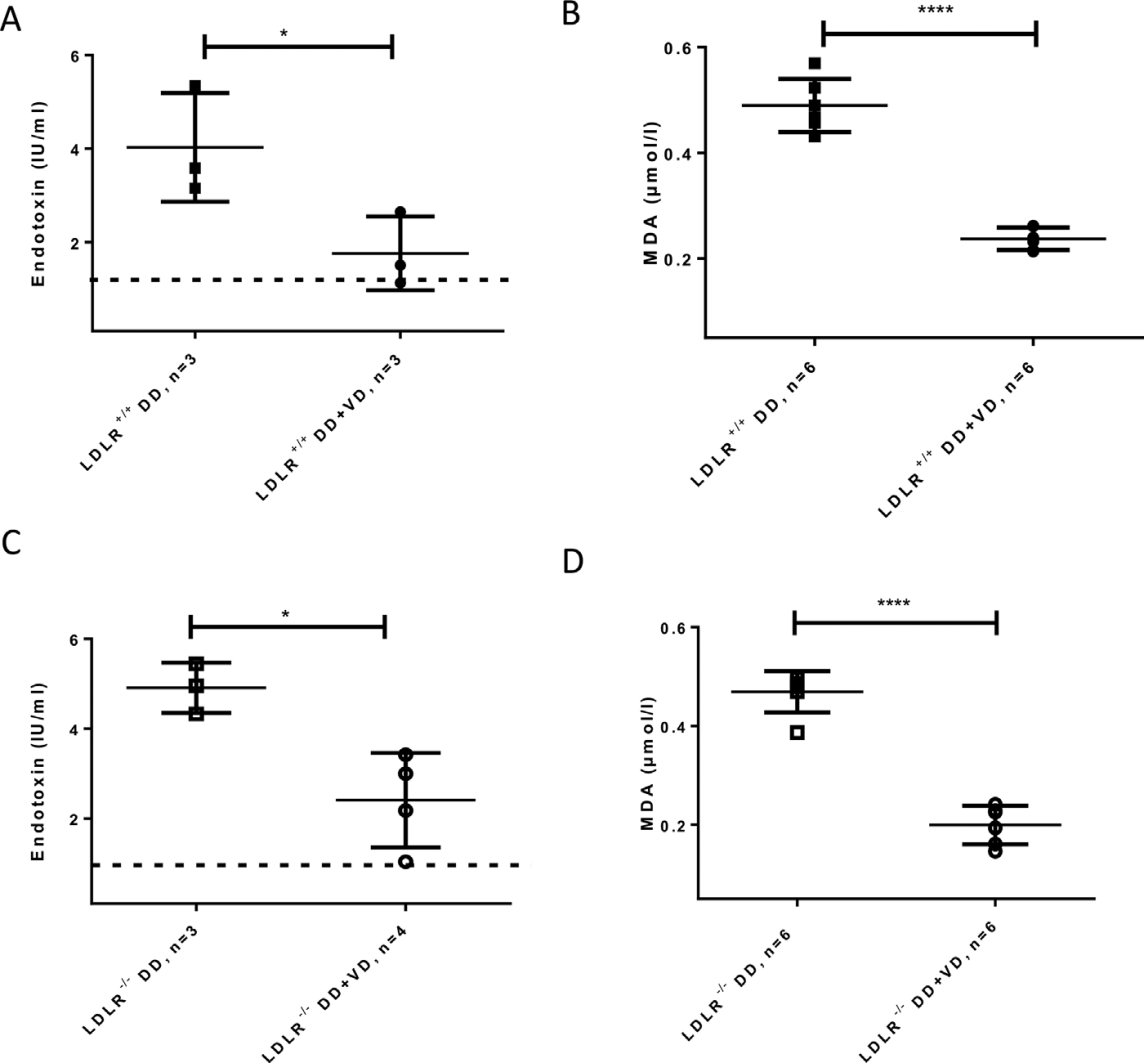
Levels of endotoxin and lipid peroxidation product, MDA, as inflammatory agents in serum of LDLR^+/+^ and LDLR^−/−^ fed a diabetogenic diet for 10 weeks without or with additional supplemented Vitamin D_3_. Dashed line in A and C represents endotoxin levels measured in mice fed the maintenance diet. *P < 0.05, ****P < 0.0001.

Endotoxaemia is a feature of obesity 18. We found that 10 weeks’ high fat high sugar diet increased endotoxin levels manifold over levels measured in mice fed a normal diet (Fig. 4A and C). Vitamin D_3_ treatment significantly decreased endotoxin levels in both groups fed a high fat high sugar diet. The Vitamin D_3_ supplemented high fat high sugar diet groups had significantly less MDA, a lipid peroxidation product, in serum than their counterparts without additional Vitamin D_3_ (Fig. 4B and D).

**Figure 5:**
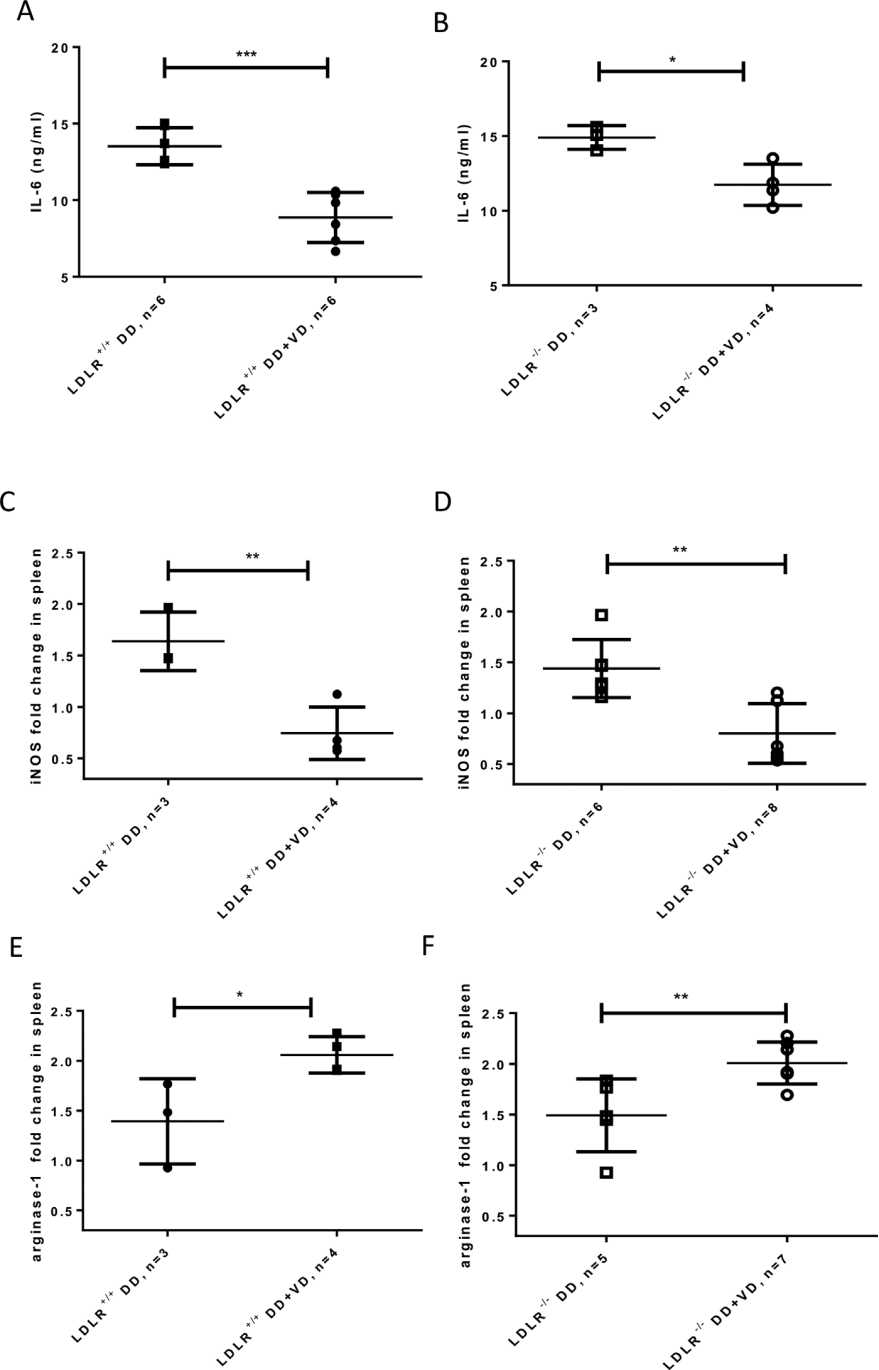
Systemic measures of inflammation and amelioration in response to additional Vitamin D_3_ supplemented to a ten weeks’ diabetogenic diet for serum IL‐6 (A and B), iNOS and arginase 1 mRNA expression in spleen (C–F). *P < 0.05, **P < 0.005, ***P < 0.0005.

Diabetogenic diets are epidemiologically linked to inflammation associated with metabolic syndrome 31, so we next assessed the impact of Vitamin D on markers of a proinflammatory profile. In the presence of additional dietary Vitamin D_3_, there was a decrease in serum levels for the hepatic master cytokine and adipocytokine, IL‐6 (Fig. 5A and B). Analysis of splenic gene expression for iNOS and arginase‐1 revealed a signature consistent with relative skewing towards M2 type mRNA expression in groups receiving additional dietary Vitamin D_3_: a decline of iNOS expression and inversely an increase of arginase‐1 expression in the Vitamin D_3_ supplemented group relative to the high fat high sugar diet group (Fig. 5C–F).

In an attempt of “Refinement” of our animal experimentation, we analyzed a shortened protocol. A significant benefit of supplementary Vitamin D_3_ was seen in LDLR^+/+^ and LDLR^−/−^ mice as early as five weeks when fed a diabetogenic diet, in terms of serum levels for IL‐6, insulin, and NEFA (Table 1). Of these mice, LDLR^+/+^ were used to investigate the impact of Vitamin D_3_ supplementation on bone mineral density. The reduced bone mineral density seen in LDLR^+/+^ mice fed a high fat high sugar diet for five weeks was normalized in those supplemented with Vitamin D_3_.

**Table 1:** Five weeks’ diabetogenic diet with or without supplementation with Vitamin D_3_

	IL‐6 (ng/ml)	Insulin (ng/ml)	NEFA (mmol/L)	Bone mineral density (mg/cc)
LDLR^+/+^ DD	1.86 ± 0.10 (*n* = 7)	1.94 ± 0.27 (*n* = 7)	0.28 ± 0.06 (*n* = 4)	2183.4 ± 18.7 (*n *= 2)
LDLR^+/+^ DD + VD	0.79 ± 0.11**** (*n* = 10)	1.21 ± 0.33*** (*n* = 10)	0.08 ± 0.02 (*n* = 5)****	2321.1 ± 34.9 (*n* = 2)* [baseline LDLR^+/+^ 2371.3 ± 39.6, *n* = 2]
LDLR^−/−^ DD	2.3 ± 0.21 (*n* = 4)	2.19 ± 0.06 (*n* = 4)	0.24 ± 0.03 (*n* = 2)	n.d.
LDLR^−/−^ DD + VD	0.75 ± 0.09**** (*n* = 5)	0.99 ± 0.41** (*n* = 4)	0.15 ± 0.01* (*n* = 2)	n.d.

Mean ± SD are indicated; ****P < 0.0001, *** *P* < 0.0005, **P < 0.005, *P < 0.05 compared to genotype matched DD group (unpaired *t*‐tests).

## Discussion

A diet high in fat and sugar is associated with a proinflammatory systemic reaction, though the exact mechanistic linkage is far less certain than the epidemiological evidence 31. Generation of oxidized LDL via free radicals, shift of gut flora to more gram negative species, translocation of LPS, and elevated free fatty acids are precipitating factors 32. Cytokine release from classically activated, so‐called M1, macrophages inhibits insulin sensitivity 33. Nutritional interventions using “food bioactives,” such as omega‐3 fatty acids, antioxidative compounds from plants or Vitamin E, are beginning to be widely tested in approaches to correct unhealthy lifestyles 34.

We analyzed female LDLR^−/−^ and LDLR^+/+^ mice on a 10 week‐Western‐style (20% protein, 36% fat, 35% carbohydrate, rich in sucrose) diet for markers of fatty liver disease, metabolic dysfunction, and inflammation. This study evaluated the effect of supplemented Vitamin D_3_ on steatosis as well as on the metabolic and inflammatory profile of the experimental mice.

LDLR^−/−^ mice were used as an aggravated model of metabolic strain to identify the scope of beneficial application of Vitamin D_3_. Their baseline levels for triglycerides and HbA1c were higher than in LDLR^+/+^. Consistent with other investigators, we have shown that mice lacking the LDL receptor, when fed a Western‐style diet, display many features of the obesity‐related metabolic syndrome, including steatosis, insulin resistance, and dyslipidemia 6. The present study has confirmed the dysmetabolic and associated hepatosteatosis changes in LDLR^−/−^ female mice fed a high fat sugar diet.

This is the first study to analyze the development of a diet‐induced prediabetic phenotype and its modulation by Vitamin D in female mice. Within a rodent high fat diet model of NAFLD, Roth et al. 35 showed that Vitamin D deficiency exacerbated histologic features of NAFLD, increased insulin resistance, and upregulated liver tissue expression of genes involved in hepatic inflammation and oxidative stress.

As previously described 28, we have demonstrated regional differences in manifestation of fatty liver. Hepatocyte lipid accumulation occurred near the periportal field in LDLR^+/+^ mice, near the central vein in LDLR^−/−^ fed a high fat sugar diet for 10 weeks. Our study finds a decrease in hepatic pericentral steatosis especially in LDLR^−/−^ mice receiving Vitamin D_3_ supplementation to their high fat high sugar diet. mRNA for Srebp‐1c, a transcription factor that regulates genes involved in lipogenesis, was significantly decreased in both, LDLR^−/−^ and LDLR^+/+^, Vitamin D_3_ supplemented high fat high sugar diet groups. In a study of male Sprague‐Dawley rats, intraperitoneal injections every other day with 1 μg/kg Vitamin D_3_ during a high fat diet (45% energy as fat) for 8 weeks led to decreased levels of triglyceride and fatty acids and improved levels of AST 36. The mechanism was thought to be inhibition of lipogenesis and promotion of fatty acid oxidation in liver, based on a decrease in Srebp‐1c mRNA and a concomitant increase of PPARα mRNA in response to injection of Vitamin D_3_. In pronounced fatty liver disease in our study, zone 3 of the hepatic lobules at the central vein, with lowest oxygen tension and harboring enzymes that regulate lipogenesis, was particularly affected. Liver transaminases are elevated in serum due to hepatocellular damage. While ALT is predominately expressed in liver, AST is more widely expressed. ALT and AST were significantly reduced to normal levels when Vitamin D_3_ was supplemented to the high fat high sugar diet. Though the diabetogenic diet was of comparable composition in the study conducted by Subramanian et al. 22, steatohepatitis and fibrotic changes were not a feature in livers obtained from our experimental animals, which is likely due to the significantly shorter duration of diet in this study (10 vs. 24 weeks).

A previous study investigating C57Bl6/J mice found no impact on body weight of dietary Vitamin D_3_, added at a comparable dose to this study 19. Others have described an effect on weight loss in mice 37, but at significantly higher dietary intake of Vitamin D_3_ (10‐fold higher from the dose chosen for this study). A role of Vitamin D_3_ in metabolism has been derived from genetically engineered mice deficient of Vitamin D receptor, in which the lack of cognate signaling led to proinflammatory alterations linked to development of insulin resistance 38. Clinical trials, however, were unable to establish a benefit of Vitamin D supplementation in enhancing insulin sensitivity 39. In our study, supplementation of a diabetogenic diet with Vitamin D_3_ led to the normalisation of insulin levels, which were pathologically elevated in those mice receiving the diabetogenic diet only. Levels of adiponectin increased reciprocally. A recent meta‐analysis of six clinical trials showed that Vitamin D supplementation for an average period of 23 weeks led to a significant increase of adiponectin levels 40. VDR and PPARγ interact 41; the *ADIPOQ* gene promoter has a PPARγ responsive element 42.

LDLR^−/−^ show a decline in the hormonally active metabolite of Vitamin D_3_ (1,25‐dihydroxy‐Vitamin D_3_) in response to 10 weeks of high fat high sugar diet. This could imply interference of the diabetogenic diet with hydroxylase activities 43 or a redistribution of the lipophilic vitamin 44. On the diabetogenic diet, LDLR^−/−^, compared to LDLR^+/+^, mice showed increased levels of triglycerides, free fatty acids and HbA1c, while endotoxin, MDA, IL‐6, and macrophage polarization were not influenced by the *Ldlr* genotype. This is consistent with LDLR^−/−^ representing a primarily metabolically strained mouse model.

This study finds, for the first time, a reversal of iNOS and arginase‐1 expression in the Vitamin D_3_ treated groups compared to the high fat high sugar diet groups, indicative of a beneficial shift towards M2 41. A certain metabolic state associates with certain macrophage activity profiles, the extremes of which are found in so‐called M1 or M2 polarization, and which may differ between adipose and other tissues. M1 type macrophages are associated with inflammation and the development of impaired cellular response to insulin, so‐called insulin resistance. M2 type macrophages are thought to predominate in the lean 45.

The gut microbiome has emerged as an important factor in development of obesity and metabolic syndrome 46. However, this study did not analyze the gut microbiome so cannot comment on the influence of a possible Vitamin D_3_ induced change in gut flora and associated endotoxin translocation 47, 48 on the observed phenotype.

Bone mineral density in LDLR^+/+^ fed a high fat high sugar diet for five weeks was significantly decreased relative to the Vitamin D_3_ supplemented group, which compared to the group on maintenance diet. Loss of bone mineral density due to a diet high in fat has previously been linked to increased osteoclast activity 49. Vitamin D, by contrast, acts by counterregulating osteoblast activity 50. However, concomitant changes in lipid oxidation products, of which MDA is a surrogate maker, are likely to impact directly on osteoblasts and osteoclasts as so‐called bioactive inflammatory lipids 51. Studies of the bone mineral density in LDLR^−/−^ fed a high fat high sugar diet are currently under way.

In conclusion, this work further supports the use of the LDL receptor knockout mouse as a useful model for studying the metabolic complications of diet‐induced obesity and insulin resistance in the presence of hyperlipidemia. In this study, Vitamin D_3_ supplementation significantly reduced the expression of the metabolic syndrome phenotype, notably progression of NAFLD, within dietary‐induced obesity among female LDLR^−/−^ and LDLR^+/+^ mice. Therefore, this study strengthens the use of Vitamin D_3_ as a so‐called nutraceutical 52—a dietary supplement with proven effect on health—because of its normalising action on lipid levels, glucose control, and systemic inflammation markers in mice fed a high fat high sugar, diabetogenic diet.

## Supporting information

Additional supporting information may be found in the online version of this article at the publisher's web‐site.

**Figure S1**. Macrovesicular steatosis (A, B and D) especially near CV (zone 3) in LDLR^−/−^ (A).

**Table S1**. Mice received diabetogenic diet and added Vitamin D_3_ (11 IU/g vs. 1 IU/g) as indicated.
**Table S2**. HbA1c levels in experimental groups; baseline indicates levels measured in LDLR^+/+^ and LDLR^−/−^ mice on normal maintenance diet.
