# Supplementary material for: Vitamin D3 supplementation of a high fat high sugar diet ameliorates prediabetic phenotype in female LDLR−/− and LDLR+/+ mice
Source: Immun Inflamm Dis. 2017 Mar 13;5(2):151–62. doi: 10.1002/iid3.154 (PMC5418139; doi:10.1002/iid3.154)
Supplement: Supplementary file 1 — Figure S1. Macrovesicular steatosis (A, B and D) especially near CV (zone 3) in LDLR−/− (A). [file IID3-5-151-s001.docx]

**C**

**A**


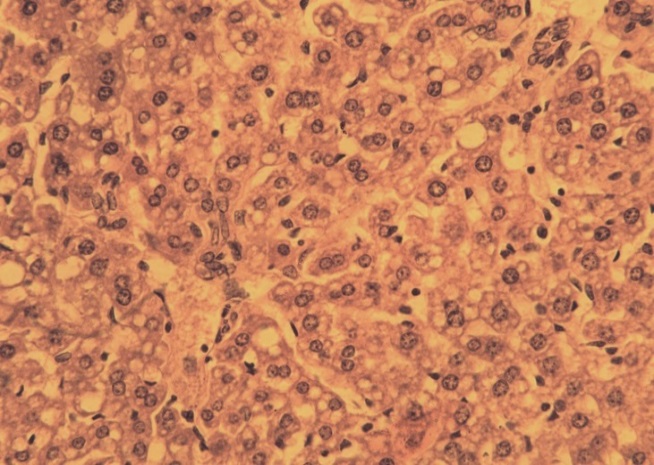


**LDLR^-/-^  DD**


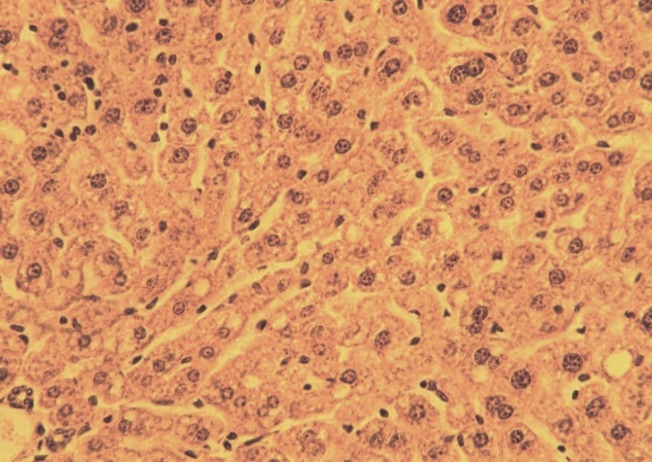


**LDLR^-/-^ DD+VD**

P

CV

CV

P

**B**

**E**

**D**


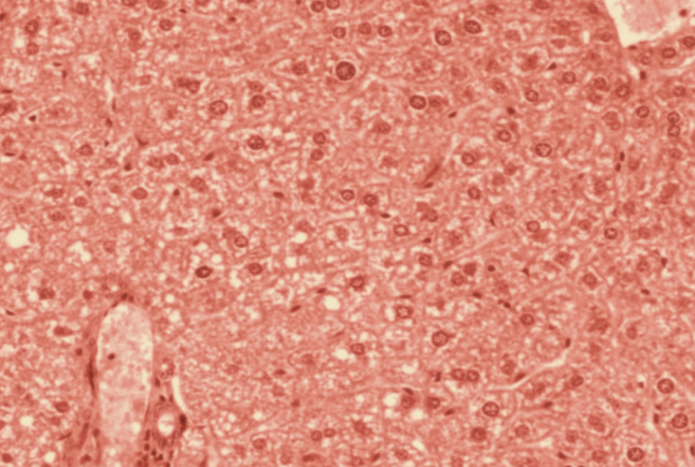


**LDLR^+/+^ DD**

CV

P


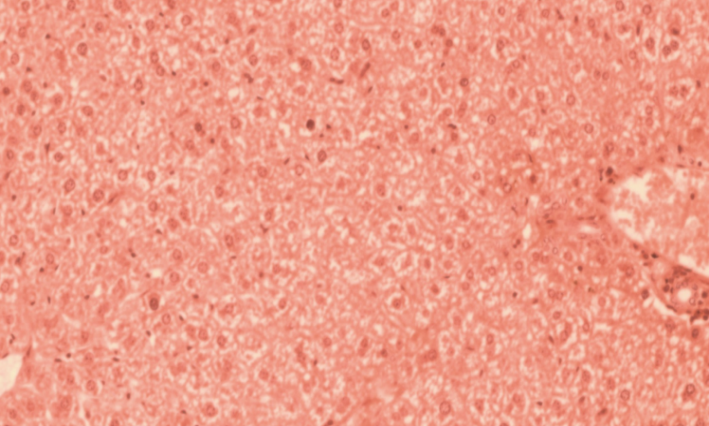


CV

P

**LDLR^+/+^ DD +VD**

**F**

Suppl. Fig 1 Macrovesicular steatosis (A,B,D) especially near CV (zone 3) in LDLR^-/-^ (A). minimal fat inclusions (E ). P, portal field CV, central vein X40 oil. Scale bar represents 100 micron; Strebp-1c mRNA expression expressed as fold change compared to GAPDH mRNA for LDLR^-/-^ (C ) and LDLR^+/+^ (F), six mice each. * p<0.05 **p<0.005
