# Supplementary material for: Vitamin D3 supplementation of a high fat high sugar diet ameliorates prediabetic phenotype in female LDLR−/− and LDLR+/+ mice
Source: Immun Inflamm Dis. 2017 Mar 13;5(2):151–62. doi: 10.1002/iid3.154 (PMC5418139; doi:10.1002/iid3.154)
Supplement: Supplementary file 2 — Table S1. Mice received diabetogenic diet and added Vitamin D3 (11 IU/g vs. 1 IU/g) as indicated. Table S2. HbA1c levels in experimental groups; baseline indicates levels measured in LDLR+/+ and LDLR−/− mice on normal maintenance diet. [file IID3-5-151-s002.docx]

| **group** | **n** | **Vitamin D_3_ (nmol/l)**  **Mean +/- SD** |
| --- | --- | --- |
| LDLR^+/+^, DD | 6 | 38.01 +/- 14.19  [baseline 48.52+/- 8.3. n=4] |
| LDLR^+/+^, DD +Vitamin D_3_ | 6 | 113.2 +/- 13.74**** |
| LDLR^-/-^, DD | 6 | 28.98 +/- 2.14  [baseline 46.21 +/- 2.8, n=4] |
| LDLR^-/-^, DD +Vitamin D_3_ | 7 | 70.12 +/- 18.0*** |

Supplementary Table 1. Mice received diabetogenic diet and added Vitamin D_3_ (11IU/g vs 1IU/g) as indicated. Baseline indicates levels measured in LDLR^+/+^ and LDLR^-/-^ mice on normal maintenance diet, 1IU/g Vitamin D_3_. Unpaired t-test Vitamin D_3_ treatment vs genotype control *** p<0.0005 **** p<0.0001

| **group** | **n** | **HbA1c (fmol/l)**  **Mean +/- SD** |
| --- | --- | --- |
| LDLR^+/+^, DD | 6 | 40.3 +/- 9.39 |
| LDLR^+/+^, DD +Vitamin D_3_ | 7 | 10.68 +/- 2.27****  [baseline 10.23 +/- 0.87, n=4] |
| LDLR^-/-^, DD | 6 | 78.98 +/- 11.72 |
| LDLR^-/-^, DD +Vitamin D_3_ | 7 | 23.26 +/- 3.14****  [baseline 14.48 +/- 2.43, n=4] |

Supplementary Table 2. HbA1c levels in experimental groups; baseline indicates levels measured in LDLR^+/+^ and LDLR^-/-^ mice on normal maintenance diet. Unpaired t-test Vitamin D_3_ treatment vs genotype control **** p<0.0001.
